# An individual-based network model to evaluate interventions for controlling pneumococcal transmission

**DOI:** 10.1186/1471-2334-8-83

**Published:** 2008-06-17

**Authors:** Diana Karlsson, Andreas Jansson, Birgitta Henriques Normark, Patric Nilsson

**Affiliations:** 1 Molecular Biology, School of Life Sciences, University of Skövde, SE-541 28 Skövde, Sweden; 2 Department of Bacteriology, Swedish Institute for Infectious Diseases Control, Karolinska Institutet, SE-171 82 Solna, Sweden; 3 Department of Microbiology, Tumor Biology, and Cell Biology, Karolinska Institutet, SE-171 77 Stockholm, Sweden

## Abstract

**Background:**

*Streptococcus pneumoniae *is a major cause of morbidity and mortality worldwide, but also a common colonizer of the upper respiratory tract. The emergence and spread of antibiotic resistant pneumococcal strains has threatened effective therapy. The long-term effects of measures aiming to limit pneumococcal spread are poorly understood. Computational modeling makes it possible to conduct virtual experiments that are impractical to perform in real life and thereby allows a more full understanding of pneumococcal epidemiology and control efforts.

**Methods:**

We have developed a contact network model to evaluate the efficacy of interventions aiming to control pneumococcal transmission. Demographic data from Sweden during the mid-2000s were employed. Analyses of the model's parameters were conducted to elucidate key determinants of pneumococcal spread. Also, scenario simulations were performed to assess candidate control measures.

**Results:**

The model made good predictions of previous findings where a correlation has been found between age and pneumococcal carriage. Of the parameters tested, group size in day-care centers was shown to be one of the most important factors for pneumococcal transmission. Consistent results were generated from the scenario simulations.

**Conclusion:**

We recommend, based on the model predictions, that strategies to control pneumococcal disease and organism transmission should include reducing the group size in day-care centers.

## Background

Infectious diseases can have a devastating impact on human life and welfare. One of the major contributors to morbidity and mortality is the bacterium *Streptococcus pneumoniae *(pneumococci). It is the main cause of respiratory tract infections such as otitis media and sinusitis, but is also responsible for millions of deaths each year due to pneumonia, meningitis, and septicemia. However, it is also a common colonizer of the upper respiratory tract, especially of children attending day-care centers (DCCs) where up to 60–70% [1,2] of the children in some studies harbor these bacteria in the nasopharynx. The effectiveness of pneumococcal treatment is hampered by the emergence of pneumococci with reduced susceptibility to antibiotics such as penicillin. Interventions have been introduced to limit the spread of pneumococci in the community. For example, decision makers have tried to promote appropriate antibiotic use [3], and to introduce pneumococcal vaccines [4,5], and to restrict day-care attendance[6]. However, the long-term effects of these measures remain to be fully evaluated. Previously, it was established that the increasing rates of antibiotic-resistant pneumococci are mainly due to the spread of strains belonging to a few number of pneumococcal clones [7].

Mathematical and computational models are invaluable tools to address epidemiological issues because they allow the conduct of virtual experiments that would be impossible or unethical to conduct in a real setting. An innovative approach is contact network modeling that captures realistic diversity in contact patterns where diseases may spread along different routes with varying degrees of transmission risk. Contact network models can be constructed on the individual-level where each individual can be assigned unique features. This enables modeling realistic heterogeneous populations and incorporation of real demographic data in the model.

Few models have addressed the question of pneumococcal transmission. Andersson *et al*[8] attempted to identify circumstances where interventions are more efficient in DCCs using a stochastic model. They concluded that interventions are more efficient in large DCC groups and during the second half of the year. Another paper, by Huang *et al*[9] reported that differences in pneumococcal prevalence across communities can partly be explained by variations in the proportion of DCC-attending children. Melegaro *et al*[10] modeled household transmission using longitudinal data of pneumococcal carriage in the United Kingdom. Their study demonstrated that, even in large families, around 50–60% of transmission occur outside the household. Hitherto, the impacts of community-wide interventions are poorly understood and need further illumination. We emphasize the importance of examining long-term effects of interventions also on the community-level to achieve more comprehensive insights into pneumococcal epidemiology. In this study we have developed an individual-based network model for simulating pneumococcal transmission, as the available models consider neither contact patterns nor structured population. The primary objective was to assess potential interventions aiming to limit the spread of pneumococci in the community.

## Methods

**Figure 1:**
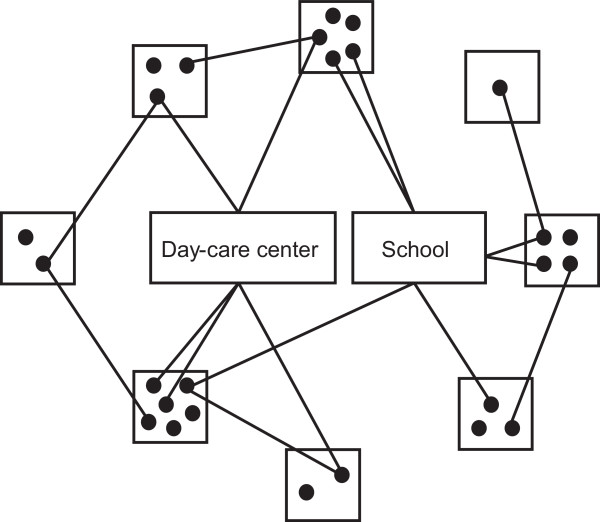
**Simplified graphic illustration of the network model concept**. The solid black dots represent individuals in the population, whereas the quadratic boxes are households, and the rectangles illustrates DCC and school, respectively. Individuals within same household have contact with each other, whereas individuals within same DCC or school have contact with each other only if they belong to the same day-care group or class. Disease transmission can only occur via edges, i.e., contacts, between vertices, i.e., individuals. The edges are bidirectional, i.e., disease may be transmitted in both directions.

Contact network models used within infectious disease epidemiology endeavor to characterize contacts occurring between individuals that might lead to transmission of diseases in the community. These contacts may take place within households, schools, DCCs, workplaces, hospitals, etc. The underlying concept of social contact networks is illustrated in Figure 1.

### Model definitions

Initially, a population consisting of 25 000 individuals was generated. Each individual in the population was assigned age according to available demographic data of Sweden [11]. All simulations were set off with identical carriers to obtain as comparable data as possible. These individuals were initially chosen based on their age to mimic observed age distribution for pneumococcal prevalence [12] allowing the simulations to start at a relatively stationary phase. Since simultaneous carriage of different serotypes is rare enough to be ignored [10], the model only considered spread of one single clone. Hence the initial number of 125 carriers corresponded to the prevalence of an arbitrary, major pneumococcal clone in Sweden [13-16]. Because the major pneumococcal clone for the time being varies, we opted to not model any particular clone. Discrete time-steps were chosen (1 week), and the update of the model was implemented to occur synchronically. The simulations were run for 780 weeks (i.e., 15 years); this relatively long running time was chosen because we were primarily interested to study the long-term effects of different interventions. Since the model is probabilistic, each run generated different results due to chance. The results of repeated runs were averaged to find the most likely outcome. As default, the simulations were executed with a fixed contact network and a static age-structure. The simulation procedures were programmed in MATLAB version 7.3.0 (The Matworks Inc.), whereas SPSS, version 15.0 (SPSS Inc.), were used for the statistical analyses.

Individuals who are colonized with pneumococci may potentially transmit the bacteria to susceptible individuals with whom they have close contacts with. For existing contacts, there is a certain transmission probability in each time step as defined by the transmission parameters (Table 1). The model did not consider progression from asymptomatic carriage to disease; only a minor proportion of the carriers develop clinical symptoms, and it is believed that the spread of pneumococci primarily occurs via asymptomatic carriers.

**Table 1:** Baseline values used for the parameters in the transmission model

**Parameter**	**Baseline value**
Transmission probability:	
Households	0.07 contact^-1 ^week^-1 ^[10]
DCCs	0.04 contact^-1 ^week^-1 ^[8]
School classes	0.03 contact^-1 ^week^-1 ^[22]
Other contacts	0.04 contact^-1 ^week^-1 ^[10]
Age-related bias:	
Individuals <1 yr	1.00 [10]
Individuals 1–6 yrs	0.33 [10]
Individuals 7–65 yrs	0.18 [10]
Individuals >65 yrs	0.3 [33]
Proportion of children attending DCCs	79% [19]
Average group size (DCC)	16.7 children [21]
Average class size (school)	22 children [22]
Probability of other contacts	0.50

### Contact sites

In network models, the structure provides features that influence the transmission of an infectious disease. Hence, only contact sites considered significant for pneumococcal spread were included in our model. These contact sites were households, DCCs, school classes and other close contacts. The contact sites can be overlapping, e.g., school children have contact both with class mates and with family members. The contact patterns were engendered by employing data of Sweden during the mid-2000s [11,17-21].

#### Households

All children 0–18 years of age were assigned 0–3 siblings according to available demographic data of Sweden for the year 2006 [18]; 19% had no siblings, 49% had one sibling, 23% had two siblings, whereas 10% of the children had three siblings or more. Due to lack of data, four children per family were assigned as maximum. Each child and his or her respective siblings were also assigned 1 or 2 parents. Parents were randomly chosen from individuals between 19–49 years of age. This age strata was chosen since the majority (~80%) of the parents with children <18 years of age are between 30 to 40 years of age according to previous studies [18]. In Sweden, about 27% of the adults live with children and 73% live without children. Of the 27% living with children, 19% are single parents and the remaining 81% of adults live as a couple with their children [18]. Of the 73% of adults who live without children, 35% live alone and the rest live as couple [18].

#### DCCs

Most (79%) preschool children in Sweden attend DCCs from 1 to 5 years of age [19]. The national mean group size, 16.7 children, was applied as default in the model [21]. The size of the day-care groups refers to the number of children in a classroom, not to the size of a DCC facility altogether.

#### School classes

In Sweden, all children between 6–15 years of age attend school. The children were assigned school class according to their age. The typical size of a class was approximately 22 children [17].

#### Other contacts

Apart from these contact sites (i.e., households, school classes, and DCCs), other close contacts occur that may transmit pneumococci such as interactions between children and their grandparents. Such other close contacts are modeled herein by contacts generated randomly. For each individual, a random contact was generated with a probability of 0.5, meaning that, on average, each individual has one other close contact. The corresponding contact was randomly chosen within the whole population. Assuming that each individual has on average one close contact, outside household, school class and DCC, seemed reasonable considering the intimacy of the contact required for pneumococcal transmission. However, in other cultures than Sweden, this assumption may appear unlikely.

### Transmission parameters and age-related biases

For an existing contact as defined by the fixed contact network, there is a certain probability in each time step for transmission to occur between individuals. In the model, this probability varied depending on contact type (Table 1). The transmission probabilities were calculated with respect to the average contact frequency occurring within a specific contact site per unit time, and also according to the intimacy of the contact type, e.g., contacts occurring between family-members are considered to be closer than contacts between class mates. Furthermore, age-related biases for susceptibility of pneumococcal colonization were used to reflect the degree of general immunity in the population. The age-related biases defined the relative susceptibilities for different age groups (Table 1).

### Duration of carriage

Large variations in the duration of pneumococcal carriage have been reported. There appears to be an inverse relationship between age and duration of carriage [6,22]. In the model, previously observed durations of carriage for respective age groups were employed [12]. An individual was assigned a carriage duration at the time point for colonization, where the duration was drawn from an exponential distribution with a mean dependent on the age of the individual (Table 2) [6,12].

**Table 2:** Mean durations of pneumococcal carriage

**Age**	**Observed mean duration (days) [12]**	**Model mean duration (weeks)**
<1 yr	74	11
1–2 yrs	47	7
3–4 yrs	34	5
5–6 yrs	26	4
7–18 yrs	26	4
>18 yrs	25	4

Observed mean durations were rounded off upwards. Since the cultures were sampled weekly, likely the observed durations to some extent are underestimated.

### Parameter analysis

To decide which of the input parameters were most important in determining the total number of pneumococcal transmission events (i.e., how sensitive the model output is to changes in the value of the parameters of the model), we performed a parameter analysis. The relationship between input parameters and the output variable were analyzed in several ways. The output variable was the number of transmission events occurring during one simulation (i.e., 780 weeks). For each parameter tested, 100 simulations were performed with varying values of the input parameter of interest, while keeping all other parameter values constant at baseline. Applying such a high number of iterations enabled us to approximate a normal distribution for the model output. At each simulation initiation, the value of the input parameter was randomly assigned from a normal distribution with a mean equal to its default value and a standard deviation (SD) of 10% of the parameter's default value. Test simulations revealed that regardless the absolute value of the SD (interval 1–10%); the relative results of the parameter analysis were likewise. However, the strength of the found relationships was stronger using a higher SD value. The parameters included in the parameter analysis are listed in Table 3.

**Table 3:** Results of the parameter analysis

**Parameter**	**Correlation coefficient, *r***	**Regression coefficient, *b***	**Elasticity coefficient, *q***
Transmission probability:			
Households	0.091 (.366)	-	-
DCCs	0.962 (.000)	2 759 932 (.000)	5.62 (5.30–5.94)
School classes	0.258 (.009)	312 819 (.000)	0.45 (0.11–0.80)
Other contacts	-0.159 (.113)	-	-
Age-related bias:			
<1 yr	-0.005 (.958)	-	-
1–2 yrs	0.882 (.000)	189 226 (.000)	3.00 (2.68–3.32)
3–4 yrs	0.841 (.000)	159 869 (.000)	2.47 (2.15–2.79)
5–6 yrs	0.680 (.000)	79 561 (.000)	1.22 (0.95–1.48)
7–18 yrs	0.343 (.000)	65 596 (.000)	0.56 (0.25–0.86)
19–65 yrs	0.169 (.093)	-	-
>65 yrs	0.103 (.307)	-	-
Duration factor	0.955 (.000)	117 047 (.000)	5.72 (5.36–6.07)
Proportion DCC attendance	0.597 (.000)	45 424 (.000)	1.79 (1.31–2.27)
Group size (DCCs)	0.948 (.000)	7 320 (.000)	5.02 (4.68–5.36)
Class size (school)	0.446 (.000)	818 (.000)	0.85 (0.51–1.19)
Probability of other contacts	0.054 (.591)	-	-

Corresponding *p*-values respective 95% CI for the coefficients are indicated in brackets where appropriate.

First, the relation between the output variable and the input parameter was examined visually by scatter plots. Then, the correlation coefficients between the output variable and the analyzed input parameters were computed using Pearson correlation to get estimates of the strength of the linear relations. Next, the effect exercised by individual parameters was examined. Regression coefficients were computed only for those parameters displaying a significant correlation (see Table 3). Because of varying dimensions of the input parameters, the regression coefficients could not be employed for direct comparison. So, we calculated the proportional effect (i.e., the elasticity):

(1) $$q=b\cdot \frac{x}{y}$$

where *q *is elasticity coefficient, and *b *is regression coefficient. The default value for the input parameter is defined by *x*, and the mean transmission events (output) are represented by *y*.

Noticeably, the parameter analysis of carriage duration was conducted by introducing a factor set to one as default. At each simulation start, the value of this duration factor was randomly assigned as described previously. During the simulations, the duration factor was multiplied by the exponentially drawn carriage duration for each newly colonized individual. Thereby, all the individual carriage durations were modified with the same factor size during a simulation.

### Intervention scenarios

It has been suggested previously that intervention strategies aiming to reduce the prevalence of pneumococci should focus primarily on the main reservoir in the community, namely children <5 years age [12,23]. Therefore, we simulated three different scenarios focusing on preschool children. The aim was to examine the consequences of i) varying the proportion of children attending day-care; ii) different sizes of day-care groups; and iii) restriction of day-care attendance for preschool children identified as carrier of pneumococci. To evaluate these candidate control measures, we first modified the contact network and then quantified the impact of these changes on the number of transmission events occurring during one simulation. Simulations of the intervention scenarios were executed 100 times for each parameter setting.

One-sample t-tests were computed for comparison of output means. The null-hypothesis considered was that there was no difference between the mean for the reference scenario and the mean for the intervention scenario.

#### Scenario 1: Varying the proportion of children attending day-care

The proportion of children attending DCCs varies between municipalities in Sweden. The national average is 79% for children 1–5 years of age [21], whereas the lowest attendance proportion is 66% (i.e., the municipality Vansbro) and the highest is 96% (i.e., the municipality Munkfors) [20]. These two extremes were employed for simulating the effect of varying proportion of day-care attendance in the community.

#### Scenario 2: Different sizes of day-care groups

During the last twenty years, the national average number of children per day-care group has increased from 13.4 (1985) to 16.7 (2006) [21]. For the years 2003 and 2004, the mean number of children per group was at its highest, 17.2. The impact of the size of the day-care groups was examined by performing simulations using 13.4 respective 17.2 children per group, on average.

#### Scenario 3: Restriction of day-care attendance for preschool children

An ongoing project in southern Sweden attempts to reduce the spread of pneumococci with a reduced susceptibility to penicillin G (MIC ≥ 0.5 mg/L) (PNSP) [6]. This is performed by an active intervention where preschool children identified as PNSP carriers are disallowed from attending DCCs until they are PNSP negative. Nevertheless, it seems impractical to identify all PNSP carriers. Therefore, we modeled this by implementing varying proportions (1%–25%) of the preschool pneumococcal carriers restricted from day-care.

## Results

### Reference scenario

**Figure 2:**
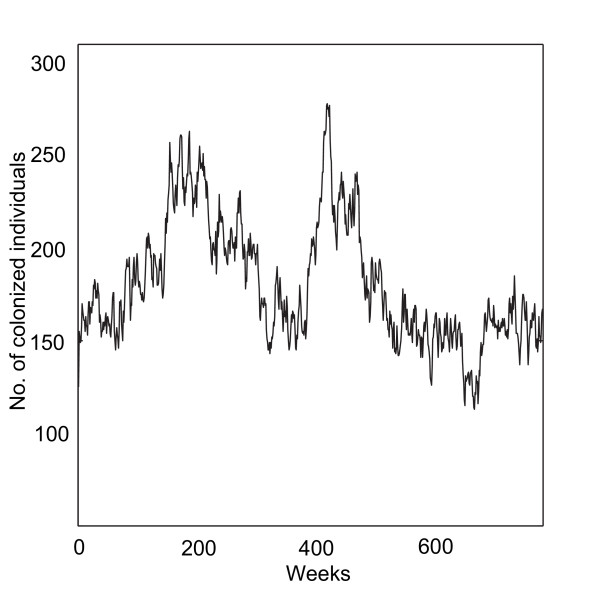
**Simulation of the reference scenario**. Example of a simulation over time using baseline parameter values given in Table 1. The number of colonized individuals fluctuates substantially in different time points (mean number of colonized individuals for all time points in 100 baseline simulations: 158.1; SD: 6.1; 95% CI: 156.9–159.3).

One hundred realizations were simulated for the reference scenario using parameter default values (Table 1). All simulations showed sustained pneumococcal prevalence. The average number of transmission events for the reference simulations was 19 318 (see additional file 1: Results of scenario simulations). An example of a baseline simulation is shown in Figure 2 where the prevalence of carriers fluctuates substantially over time.

**Figure 3:**
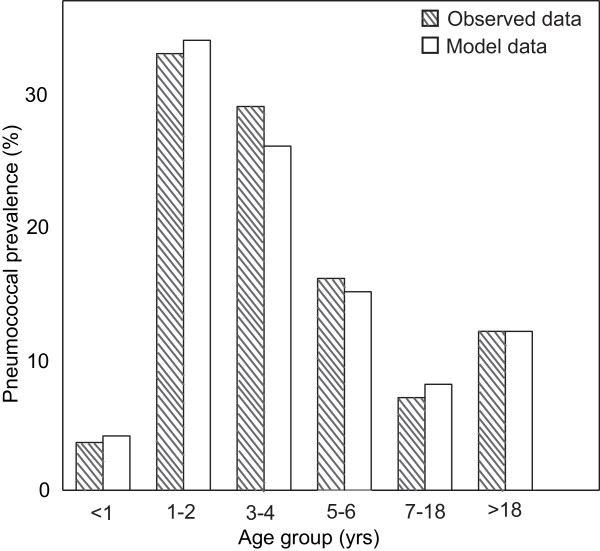
**Age distribution for prevalence of pneumococcal carriage**. The distribution for prevalence of pneumococcal carriage in different age groups resulted from the model simulations compared with observed data. From Högberg *et al*. (2007) [12] observed data are derived, whereas model data are the mean values of 100 baseline simulations (Age group < 1 yr: mean 4.1%; 95% CI 3.9–4.2%; Age group 1–2 yrs: mean 34.0%, 95% CI 33.2–35.2%; Age group 3–4 yrs: mean 26.5%, 95% CI 25.7–27.2%; Age group 5–6 yrs: 15.4%, 95% CI 14.9–15.9%; Age group 7–18 yrs: mean 8.0%, 95% CI 7.8–8.3%; Age group >18 yrs: 11.9%, 95% CI 9.8–13.9%).

The age distribution among the carriers resulting from the reference simulations was compared with existing data [12]. The data used for comparison was the observed mean durations of nasopharyngeal carriage of PNSP, as we modeled the spread of an arbitrary pneumococcal clone. Since prevalence is a composite of both incidence and duration of carriage, the numbers of transmission events generated by the model were adjusted for mean durations of carriage for each specific age group. The number of transmission events occurring within each age group were multiplied with the expected duration, i.e., observed mean duration (Table 2). Next, the prevalence for each age group was calculated as the fraction of the total number of colonization weeks. The age distribution of prevalence resulted from the baseline simulations corresponded well with observed carriage prevalence in Sweden (Figure 3).

More than half of all transmissions (66%) occurred within day-care groups supporting the notion that DCCs are the most important environments for the spread of pneumococci. Also a significant part of the transmissions occurred within households (21%), whereas only a minority of the pneumococcal spread came about in schools (8.8%) and via other contacts in the society (4.2%), according to the model.

### Parameter analysis

**Figure 4:**
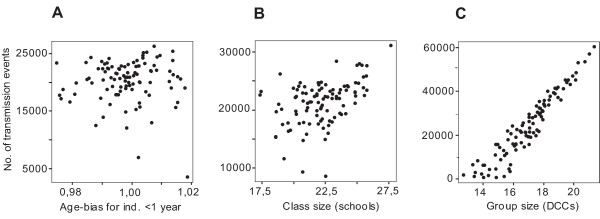
**Scatter plots exemplifying three correlation outcomes**. A. Scatter plot for the parameter *AgeBias <1 yr *with correlation coefficient *r *= 0.005 (Table 3). Correlation coefficients near 0 indicate weak, negligible relationships. B. Corresponding scatter plot for the parameter *Class size *(school) (*r *= 0.446) is shown. C. Scatter plot showing a strong, positive linear relationship. The parameter analyzed is *Group size *(DCCs) with correlation coefficient *r *= 0.948.

In the parameter analysis, sixteen parameters were included (Table 3). More than half of these displayed a significant correlation. Some examples of typical outcomes are shown as scatter plots in Figure 4. The regression coefficients were used for calculation of the elasticity coefficients for the parameters. The elasticity analysis suggested that *Duration factor, Day-care transmission*, and *Group size (DCC) *as the parameters exerting most influence on the spread of pneumococci.

### Intervention scenarios

The intervention scenarios were compared with the reference scenario to evaluate the simulation results.

#### Scenario 1: Varying the proportion of children attending day-care

We ran two scenarios simulating the significance of the community proportion of children attending DCC. In Sweden, the lowest DCC attendance in a municipality is 66% [20]. Using this low extreme resulted in a reduction of the mean number of transmission events by 16% (see additional file 1: Results of scenario simulations). When simulating a high day-care attendance, we used the highest DCC proportion in Sweden, namely 96% [20], which resulted in raising mean transmission events by 55%.

#### Scenario 2: Different sizes of day-care groups

The size of the day-care groups were examined by first modeling a small group size, 13.4 children per group and thereafter a larger consisting of 17.2 children per group. Mean transmission events of simulations for small group size dramatically declined by 82% (see additional file 1: Results of scenario simulations), whereas the mean for large group size increased by 20%, compared with the reference scenario.

#### Scenario 3: Restriction of day-care attendance for preschool children

Varying the percentage of carriers restricted from attending DCCs were used for simulating an active intervention of pneumococcal transmission. Unexpectedly, the simulations showed a significantly reduction in prevalence of pneumococci already when 1% of the children carrying pneumococci at the DCCs were disallowed attendance at the DCCs (see additional file 1: Results of scenario simulations), and mean transmission events were lowered by 12%. The corresponding reduction in mean transmission events for 5% and 10% isolation were 41% and 63%, respectively. Simulations with 15% isolation lowered the mean transmission events by 82%.

## Discussion

We constructed an individual-based probabilistic network model to gain increased insight into the epidemiology and spread of *S. pneumoniae*. In our approach, the transmission model of asymptomatic pneumococcal carriage was embedded in a demographic model describing Sweden in the mid-2000s. A network structure was engendered through individuals attending different contact sites such as households, DCCs, school classes and other close contacts occurring in the society. In addition, the individuals were assigned an age-biased susceptibility of pneumococcal colonization. The baseline model was able to reproduce observed age-distribution for prevalence of pneumococcal carriage in Sweden relatively well (Figure 3). The primary aim was, however, to reveal key determinants in transmission of pneumococci and thereby feasible targets for interventional actions. To achieve this, we analyzed several model parameters by investigating the individual parameter's quantitative impact on pneumococcal incidence. The parameter analysis revealed three features as more influential than others i) the durations of carriage; ii) the transmission probability within DCCs, and iii) the group size in the DCCs.

Thus, the duration of carriage was shown to be of most relevance for pneumococcal spread. Duration of carriage has been observed to vary with pneumococcal serotype [12] and are also to be influenced by host-related factors. The duration of carriage is a factor that is hard to influence, and hence is difficult to consider as an interventional alternative. On the other hand, this finding can give us insight into why certain pneumococcal clones are more successful in their spread than others, besides bacterial features such as expression of adhesive pili as previously reported [7]. Also the probability of transmission within DCCs was elucidated as a key determinant in pneumococcal transmission. Ways to lower the DCC transmissibility could for example be by improved hygiene routines.

The prevalence of pneumococcal carriage varies widely across communities in the United States [9]. The proportion of children who attend DCCs has been suggested to account for a range of 4%–56% in the prevalence of carriage [9]. However, our model results indicated that the size of the DCCs groups is a factor that may have even greater impact on pneumococcal incidence. By reducing the size of the DCCs groups, e.g., by increasing the number of classrooms without reducing the proportion of children attending DCCs, on a community-wide basis, pneumococcal spread can be reduced markedly according to simulations of the model (see additional file 1: Results of scenario simulations). Previously, it has been reported that the pneumococcal carriage rate is significantly higher in DCC facilities attended by more than or equal to 45 children, than in centers with less than 45 children [24].

A disadvantage of the intervention strategy of reducing the sizes of the DCCs groups is increased costs for the society by way of introduction. However, we speculate that these costs may be compensated on long-term by reduced prevalence of other infectious agents besides pneumococci in the community, and this would probably result in lower absence due to sickness and a decreased need of medical resources.

The parameter defining pneumococcal susceptibility in the model, *Age-related bias*, varied with age and was analyzed by splitting the population into seven age groups (Table 3). This parameter was considered as a relative susceptibility factor, a higher value generated a higher probability for colonization when exposed to pneumococci. One way to lower age-related biases is for example by vaccination of certain risk groups. The age-bias for 1–2 years was discerned as most significant; consequently, vaccination of children 1–2 years of age would be most effective. Only conjugate vaccines have so far been shown to be protective in this age group [25,26]. However, these vaccines only include a limited number of 7–13 capsular types of the 91 serotypes. Additionally there is likely some increase, albeit small, in the rate of non-vaccine serotypes as a result of vaccine use [25,26]. The effects of pneumococcal vaccines on nasopharyngeal carriage have been investigated previously. Studies on conjugate vaccines have reported a reduction in vaccine-type colonization and a concomitant increase in non-vaccine type carriage [27-29], whereas polysaccharide vaccines seems to have no effect on pneumococcal colonization [30]. The model described in this paper is, however, unable to examine such effects of vaccines. Research on pneumococcal vaccines that may cover all pneumococcal types, including protein-based vaccines is ongoing. For the future, vaccination offers promising potential for reducing pneumococcal carriage and thus transmission.

Time simulations demonstrated that the prevalence of pneumococcal colonization varies in different time points (Figure 2). This sort of rapid fluctuations in prevalence of different pneumococcal clones has been reported in previous studies [31]. Our results indicate that this dynamic is inherent, and is likely a result of the network structure of contact patterns in combination with probability factor. Consequently, the observed fluctuations in prevalence of pneumococcal clones do not necessarily have to be caused by antibiotic selection or herd immunity as suggested previously [23].

According to our scenario simulations, the DCC group size seems to play an important role in pneumococcal transmission. By decreasing the average DCC group size from 16.7 to 13.4 children, the model predicts a reduction in transmission of 82%. Simulations conducted where the average DCC group size was reduced below 13.4 children (data not shown) showed likewise results as when more than 15% of the pneumococcal carriers were denied attending day-care (see additional file 1: Results of scenario simulations). The number of transmission events occurring are substantially decreased, and besides, for the majority of the simulations the pneumococcal prevalence is not sustained in the *in silico *population. Conversely, a decrease in the community proportion of children attending DCCs seems to have only a small effect on pneumococcal spread. The reduction in transmission events observed when simulating 66% of children attending DCCs is in line with what happens if 1% of the children carrying pneumococci are kept home and not allowed to attend the DCCs. A relatively high density within DCC groups combined with that individuals attending DCCs have relatively high susceptibilities (*Age-related bias*) and long durations of carriage in the model, explains how whether decreasing the average DCC group size or restricting pneumococcal carriers from attending day-care have such an effect it does in the model.

Simulations indicated that at least 15% of the DCC-attending carriers need to be kept at home to achieve an equivalent effect as when reducing the sizes of the DCC groups. Even if it seems to be an effective intervention to not allow carriers to attend the DCCs, it will bring considerable costs for the society and for the individual households. Isolation of a PNSP-carrying child requires one parent to stay at home from work for an appreciable period, up to nearly 1 year in some cases [6,12,32].

This transmission model has several limitations. It does not account for variations in transmissibility due to differences in DCCs attendance (hours), though it has been suggested previously that it may be an important factor [9]. However, we speculate that the number of hours spent in a DCC may be more relevant in models considering DCCs as the only environments for spread. Another shortcoming may be that we assumed general age-dependent immunity-levels in the modeled population. Since it is unclear whether immunity develops or not after a carriage event, this assumption in seemed the most appropriate. Furthermore, the advantages of using an individual-based model structure may not be exploited fully in this study; for example we used neither a dynamic age-structure nor considered individual histories of pneumococcal exposures. However, this initial model may serve as a platform for future, more complex models for addressing other epidemiological issues.

## Conclusion

In conclusion, our results suggest that among the scenarios considered after running intervention strategies for 15 years, the most efficient way to decrease pneumococcal carriage and spread in the community is to reduce group sizes in the DCCs. By combining decreased DCC group size with other interventional actions, the effect may be strengthened. The model developed in this paper can be used for further studies of the epidemiology of pneumococcal spread and the consequences of vaccination of certain risk groups including possible herd immunity effects.

## Competing interests

The authors declare that they have no competing interests.

## Authors' contributions

DK carried out the majority of the model design and construction, and the model implementation, all analyses, interpreted the results and prepared the manuscript as the lead writer. AJ participated in the model design, the model implementation and helped to draft the manuscript. BHN participated in the model design and helped to draft the manuscript. PN participated in the model design and helped to draft the manuscript. All authors read and approved the final manuscript.

## Pre-publication history

The pre-publication history for this paper can be accessed here:



## Supplementary Material

Additional file 1 Results of scenario simulations. Table with the results of the scenario simulations. Click here for file
