# Supplementary material for: An individual-based network model to evaluate interventions for controlling pneumococcal transmission
Source: BMC Infect Dis. 2008 Jun 17;8:83. doi: 10.1186/1471-2334-8-83 (PMC2442080; doi:10.1186/1471-2334-8-83)
Supplement: Additional file 1 — Results of scenario simulations. Table with the results of the scenario simulations. [file 1471-2334-8-83-S1.doc]

| **Scenario description** | **Mean output^*^** | **Percentage of simulations with sustained prevalence** | **Standard**  **deviation** | **Mean difference^†^** | **Maximum**  **output^‡^** | **Minimum output^‡^** |
| --- | --- | --- | --- | --- | --- | --- |
| Reference scenario (79% DCC attendance; 16.7 children/group) | 19 318 | 100 | 2 860 | - | 26 415 | 9 660 |
| Proportion children attending DCC: |  |  |  |  |  |  |
| 66% | 16 304 | 99 | 3 294 | -3 014 | 21 992 | 4 634 |
| 96% | 29 909 | 100 | 3 307 | 10 591 | 36 726 | 22 783 |
| Average DCC group size: |  |  |  |  |  |  |
| 13.4 children | 3 488 | 38 | 1 772 | -15 830 | 8 104 | 537 |
| 17.6 children | 23 109 | 100 | 3 139 | 3 791 | 28 936 | 14 449 |
| Proportion of children isolated from DCC: |  |  |  |  |  |  |
| 1% | 17 054 | 100 | 3 294 | -2 264 | 24 438 | 8 510 |
| 5% | 11 493 | 97 | 3 396 | -7 825 | 17 932 | 704 |
| 10% | 7 087 | 71 | 3 115 | -12 231 | 13 419 | 898 |
| 15% | 3 442 | 27 | 1 953 | -15 876 | 8 398 | 644 |
| 25% | 1 193 | 1 | 531 | -18 125 | 3 615 | 456 |
| ^*^Mean outputs refer to the mean number of transmission events for 100 simulations of each scenario. Mean outputs for the intervention scenarios were compared with the mean output for the reference scenario, and all mean outputs were significantly changed with *p*<0.001.  ^†^Mean difference when comparing the mean transmission events of scenario simulations with mean transmission events of the reference simulations.  ^‡^Maximum and minimum outputs refer to, respectively, the highest and the lowest number of transmission events that occurred during a single simulation of the specified scenario. | | | | | | |
